# Aeruginosin 525 (AER525) from Cyanobacterium *Aphanizomenon* Sp. (KUCC C2): A New Serine Proteases Inhibitor

**DOI:** 10.3390/md22110506

**Published:** 2024-11-08

**Authors:** Donata Overlingė, Marta Cegłowska, Robert Konkel, Hanna Mazur-Marzec

**Affiliations:** 1 Marine Research Institute, Klaipėda University, Universiteto av. 17, LT-92294 Klaipėda, Lithuania; 2 Institute of Oceanology, Polish Academy of Sciences, Powstańców Warszawy 55, PL-81712 Sopot, Poland; mceglowska@iopan.pl; 3 Department of Marine Biology and Biotechnology, University of Gdańsk, M. J. Piłsudskiego 46, PL-81378 Gdynia, Poland; robert.konkel@ug.edu.pl (R.K.); hanna.mazur-marzec@ug.edu.pl (H.M.-M.)

**Keywords:** enzyme inhibition, bioactive peptides, high-resolution mass spectroscopy

## Abstract

Aeruginosins (AERs) are one of the most common classes of cyanobacterial peptides synthesised through a hybrid non-ribosomal peptide synthase/polyketide synthase pathway. They have been found in *Microcystis*, *Nodularia spumigena*, *Oscillatoria*/*Plantothrix*, and *Nostoc.* The presence of AER in *Aphanizomenon* isolated from the Curonian Lagoon was reported for the first time in our previous work. Here, the structure of aeruginosin 525 (AER525), isolated from *Aphanizomenon* sp. KUCC C2, was characterised based on high-resolution mass spectrometry. This new AER variant shows potent activity against thrombin. It also inhibits trypsin and carboxypeptidase A but has no effect on elastase and chymotrypsin. In terms of the *N*-terminal residue and biological activity, AER525 displaces some similarity to dysinosins, which belongs to the most potent inhibitors of thrombin among AERs. The findings underline the potential of AER525 as a new anticoagulant agent.

## 1. Introduction

*Aphanizomenon* is a prokaryotic cyanobacterium that occurs worldwide in fresh [1] and brackish aquatic ecosystems [2]. Due to its high content of proteins, essential amino acids, vitamins, and minerals, this genus has been used for human consumption for many years [3]. The Upper Klamath Lake in North America, which is the primary production source of *Aphanizomenon* biomass, is the only place where these cyanobacteria are grown in the wild. Food supplements from this aquaculture are produced on an industrial scale and distributed worldwide [1,3]. Cyanobacteria of this genus are one of the most important sources of food supplements (second after *Spirulina*) [4]. However, only a few studies have explored the properties of *Aphanizomenon* from batch cultures. So far, its mass cultivation has not been attempted or achieved, and the requirements for optimal *Aphanizomenon* growth have not been defined [5,6,7]. Its biotechnological potential has also not been fully recognised [5].

In addition to the nutritional properties of *Aphanizomenon*, assessed mainly based on the analyses of extracts, there are several studies showing that the cyanobacterium produces metabolites of significant biological activities. These include anabaenopeptins I and J, potent carboxypeptidase-A inhibitors detected in the NIES-81 strain [8], and trypsin inhibitors, aphapeptin F1 and F2 isolated from the 008a and X0023 strains [9]. Other metabolites, β-phenylethylamine and phycocyanobilin isolated from Klamath Lake *Aphanizomenon*, showed effectiveness in alleviating depression and anxiety symptoms [10,11] and acted as inhibitors of the human cytosolic enzyme which is involved both in tumor progression and phytochemical bioavailability [12]. Additionally, extracellular polymeric substances produced by this cyanobacterium induced cell cycle arrest of human A431 epidermoid carcinoma cells through the mitochondrial pathway [13]. In our previous studies, we documented antibacterial activity and inhibition of serine protease by two *Aphanizomenon* strains from the Curonian Lagoon. In crude extracts from these cyanobacteria, several anabaenopeptins and aeruginosin were detected [14].

Aeruginosins (AERs) are a class of linear peptides biosynthesised by cyanobacteria through a hybrid non-ribosomal peptide synthase/polyketide synthase pathway [15]. They exhibit inhibitory activity against serine proteases [16,17]. The first AER (298A) was isolated in early 1994 by Murakami [18] and co-workers from *Microcystis aeruginosa*. The peptide affected the activity of thrombin, trypsin, and plasmin [18]. To date, 90 AERs or AER-like compounds have been described, of which 2 are synthetic [19,20]. Their high structural diversity could be attributed to various recombination processes affecting AER biosynthesis (e.g., acquisition of accessory enzymes, large-range recombination of AER biosynthesis genes) [21]. As a consequence, different residues with a wide variety of functional groups can be found in the AERs’ structures. The common feature of all AERs is the 2-carboxy-6-hydroxy-octahydroindole (Choi) residue. Possible modifications in the structure of AERs include glycosylation, ethylation, bromination, chlorination, sulfation, and epimerisation [22]. Several closely related compounds, such as dysinosin, desoxydysinosin, chlorodynosin, oscillatoxin, microcin, and suomilide, share structural similarities with AERs and are classified to the same family [16,23,24,25,26,27,28,29]. All known AER variants were isolated from *Microcytis* bloom samples, cyanobacterial cultures of *Nodularia spumigena*, *Microcystis*, *Oscillatoria*/*Plantothrix*, *Nostoc*, sponges, or their cyanobacterial symbiont *Hormoscilla spongeliae* (e.g., refs. [23,30,31,32,33,34,35,36]).

*Aphanizomenon* bloom is an annual phenomenon recorded in the Curonian Lagoon, located in the southeastern part of the Baltic Sea [37,38,39,40]. Until now, studies of the biotechnological potential of *Aphanizomenon* from this region have been mainly based on bloom samples collected during the dominance of the genus. Several studies found that extracts from bloom samples were rich in α-tocopherol and α-linolenic acid and had antioxidant properties [41]. Additionally, extracts from this cyanobacterium, rich in phycobiliproteins, showed oxidative properties and a cytotoxic effect against C6 glioblastoma cells [42]. Phycocyanins extracted from the biomass dominated by *Aphanizomenon flos-aquae* were tested for their stability and applications for value-added products [43]. Furthermore, Overlingė et al. [14,44] showed that extracts obtained from bloom samples dominated by *Aphanizomenon* and extracts from isolated *Aphanizomenon* strains, KUCC C1 and KUCC C2, had antibacterial and enzyme inhibitory activities. This activity of *Aphanizomenon* KUCC C2 was further explored in the current work. As an active agent, a new AER variant, AER525, was identified. In the latest version of the CyanoMetDB [19,20], there is no compound with a molecular weight of AER525, indicating that it is a new aeruginosin variant.

Although AERs with partially elucidated structures were detected during *Aphanizomenon* blooms in the Curonian Lagoon [40,44,45], to our knowledge, no studies have been published on the biological activity of pure compounds isolated from this genus. Furthermore, there is no documented evidence of AER production by *Ahanizomenon*. Therefore, the main aim of this work was to explore the chemical structure and effects of the isolated AER525 on serine proteases and carboxypeptidase A.

## 2. Results

### 2.1. Isolation and Structure Characterisation of AER525

**Figure 1:**
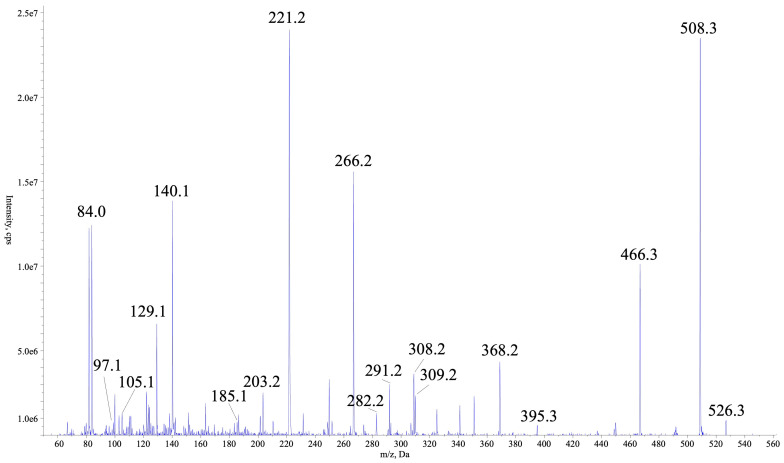
Mass fragmentation spectra of AER525 collected using QTRAP5500.

**Figure 2:**
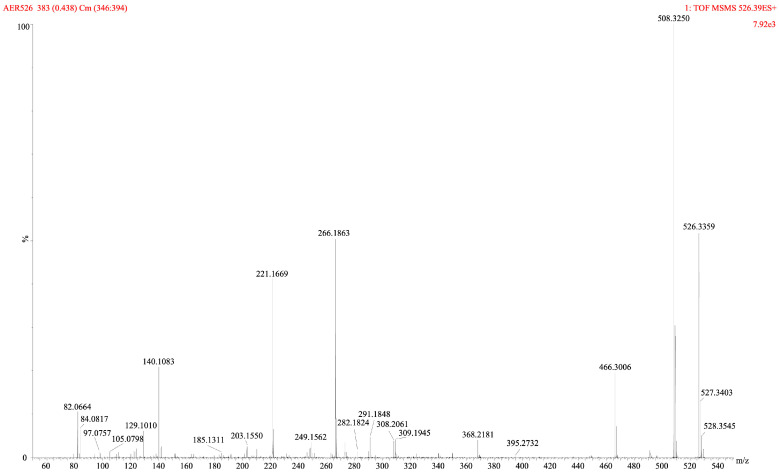
Mass fragmentation spectra of AER525 collected using HRMS SYNAPT XS QTOF systems.

To identify the compound responsible for enzyme inhibition observed in the assays with crude KUCC C2 extract [14], bioassay-guided fractionation was performed. Fractions obtained from the active extract were analysed with LC–MS/MS system in untargeted mode and tested against trypsin and thrombin. The unidentified compounds detected in fractions F0, F2–F4, which inhibited the enzymes by more than 50%, are listed in Table S1. In fractions F24–F25, AER with pseudomolecular ion at *m*/*z* 526 was detected as the main component (Table S2). From these two fractions, AER525 was isolated. In the rich fragmentation spectrum collected in the enhanced product ion mode (EPI; QTRAP5500), a series of ions confirming the presence of Leu* + Choi + Argal fragments were detected (Figure 1). Argal (argininal) is an arginine (Arg) mimetic with an aldehyde group instead of a carboxylic group. The high-resolution TOF MS (SYNAPT SX) gave a spectrum with a similar fragmentation pattern and all ions crucial for structure elucidation (Figure 2, Table 1). In the total ion chromatogram (TIC), the precursor ion with *m*/*z* 526 gave peaks with different retention times, but identical fragmentation patterns, suggesting the occurrence of AER525 isomers (tautomers) (Figure S1). The general formula of AER525 (C_24_H_43_N_7_O_6,_ exact *m*/*z* 526.3353) was determined based on HRMS analyses and the spectrum with [M+H]^+^ ion at 526.3359 (Δ = 0.0006). The collected data indicate that in the *N*-terminal position of AER525 is a residue with general formula C_3_H_6_O_2_N and exact mass 88.0398 is present. This corresponds to the 2-amino-3-hydroxy-propionic acid residue (Ser) or 3-amino-2-hydroxy-propionic acid (Figure 2). Based on MS analyses, the structural isomers of Leu (Ile) and Ser (3-amino-2-hydroxy propionic acid) cannot be distinguished here, they are marked with asterisk (Leu* and Ser*).

In the TIC chromatogram of isolated AER525, an ion with *m*/*z* 643.4146 was also detected (Figure S2). This ion occurred at exactly the same retention time as AER525 and showed the same fragmentation pattern. Initially, we assumed that either *Aphanizomenon* KUCC C2 produces another AER variant with a similar structure or that this ion is formed during a reaction that takes place in the ion source. However, the peptide with *m*/*z* 643 was not detected when direct injection of the sample into the ion source of the HRMS was applied. Based on these results, we finally concluded that the peptide is an artifact formed in the column, during chromatographic separation. This case illustrates the need for careful assessment of collected MS data, before further processing and interpretation is performed; especially, when MS data are applied in the molecular networking of natural products.

### 2.2. Enzymatic Assay of Aphanizomenon Sp. KUCC C2 Fractions

**Figure 3:**
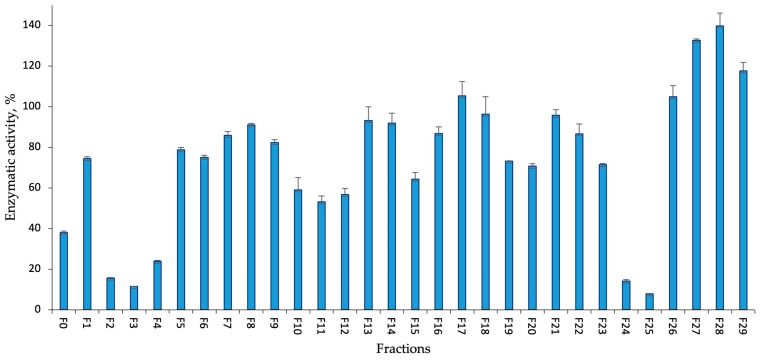
The activity of *Aphanizomenon* sp. KUCC C2 fractions against trypsin (tested at a concentration of 45 µg mL^−1^).

Among the flash chromatography fractions obtained from the KUCC C2 extract and tested against trypsin (at a concentration of 45 µg mL^−1^), six fractions showed inhibition of the enzyme by more than 60% (Figure 3). Four of them were eluted with the lowest methanol concentration (F0, F2–F4) and two were eluted with 95% methanol (F24–F25). The most potent activity was recorded for fractions F2, F3, F4, F24, and F25 where the enzyme activity was reduced by 84, 88, 76, 86, and 92%, respectively.

**Figure 4:**
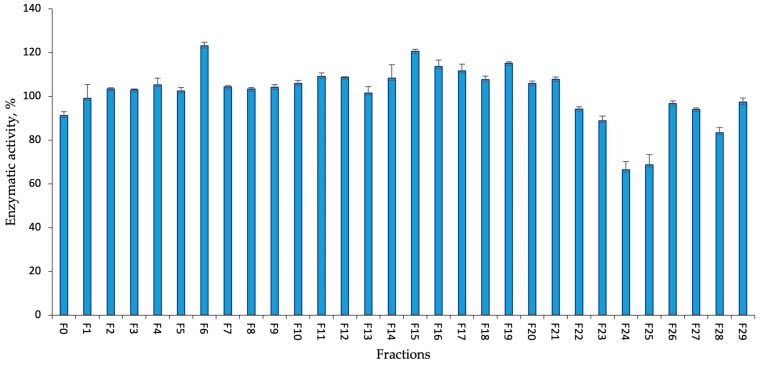
The activity of *Aphanizomenon* sp. KUCC C2 fractions against thrombin (tested at a concentration of 45 µg mL^−1^).

The activity of the KUCC C2 flash chromatography fractions against thrombin was moderate (Figure 4). Only two fractions, F24 and F25, had some effect on the enzyme, reducing its activity by 34% and 31% of the untreated sample, respectively.

### 2.3. Enzymatic Assay of AER525

The isolated AER525 was tested against 5 enzymes—trypsin, thrombin, carboxypeptidase A, chymotrypsin, and elastase (Table 2). No activity against chymotrypsin and elastase was observed. AER525 had the strongest effect on all other enzymes at a concentration of 45 µg mL^−1^, reducing trypsin activity by 60%, thrombin by 80%, and carboxypeptidase A by 47%. The IC_50_ value for trypsin was 71.71 µM, thrombin 0.59 µM, and carboxypeptidase A 89.68 µM.

## 3. Discussion

In our previous study, the metabolic profile and biological activities of two *Aphanizomenon* strains from the Curonian Lagoon, KUCC C1 and KUCC C2, were compared [14]. Extracts from both strains inhibited trypsin and thrombin, but only in KUCC C2 aeruginosin, the compound representing a known class of enzyme inhibitors was detected.

The linear peptides, aeruginosins, are produced by cyanobacteria through the action of a hybrid non-ribosomal peptide synthetase (NRPS)/polyketide synthase (PKS) enzyme complexes [15]. NRPS and PKS have a modular structure and each module catalyses the incorporation of a specific amino acid or another residue in the structure of the peptide. The most characteristic feature of AERs’ structure is the presence of Choi and *C*-terminal Arg or its mimetics. The second position is occupied by hydrophobic amino acids, with Leu/Ile being the most frequent [19]. In the majority of AERs, the *N*-terminal position is occupied by hydroxyphenyllactic acid (Hpla) or phenyllactic acid (Pla) [15,17]. AER-like compounds, dysinosins and pseudoaeruginosins, are biosynthesised through pathways closely related to AERs. The unique trait of pseudoaeruginosins is the presence of *N*-terminal short fatty acid chains [30], while in dysinosins this position is occupied by sulfated glyceric acid (with possible modifications) [23,36]. In dysinosin D, the sulfate group is replaced by the hydroxyl group. The presence of 2-amino-3-hydroxy-propionic acid (Ser) or its structural isomer at the *N*-terminus of AER is reported in our work for the first time. This residue differs from the *N*-terminal group in dysinosin D by the presence of an amine group, instead of the OMe (Figure S3). In the current study, the presence of 2-amino-3-hydroxy-propionic acid (or its structural isomers) was deduced based on the spectra collected in enhanced product ion scan (QTRAP) and ion mobility experiments (SYNAPT XS). The exact mass of the peptide was determined with a high-resolution Time-of-Flight MS. Unfortunately, despite repeated chromatographic runs performed under optimised conditions, the attempts to isolate sufficient amounts of the peptide for NMR were not successful. The difficulties were due to the tautomerisation process of the *C*-terminal Argal present in AER525. The problem generated by the tautomerisation of Argal, which also hampers the NMR analyses, was addressed by other authors who studied the structure and bioactivity of AERs (e.g., refs. [46,47]). During chromatographic separations performed in our work, AER525 eluted at different retention times (Figure S1). A similar pattern was observed by Entfellner et al. [21], who analysed AER variants synthesised by the *Planktothrix* strain No66. The compounds exhibited identical protonated masses and fragmentation profiles, but eluted at different retention times, suggesting the presence of isomeric forms.

With respect to the short acid chain in the *N*-terminal position, AER525 is structurally similar to dysinosin D (Figure S3). Both peptides inhibit thrombin with comparable potency. Dysinosins (A–D) inhibit factor VIIa and thrombin, the two enzymes involved in the blood coagulation cascade [23,36]. However, dysinosins A–C are 10 times more active than dysinosin D [23]. This difference in activity is attributed to the presence of the sulfate group in the *N*-terminal glyceric acid of dysinosins A–C, while this group is lacking in dysinosin D [23]. AER525, isolated from KUCC C2, exhibits a distinctive structure and strong thrombin-inhibitory activity, positioning it as a promising candidate for further investigation of its possible pharmaceutical potential. However, as the progress of the research is limited by the amounts of isolated AER525, an alternative method of its production should be elaborated. One option is chemical synthesis, which can also be used to optimise the structure and drug-like properties of the compound.

Due to the high chemical diversity of AERs and their chemical modifications, they have different inhibitory activity against enzymes and have an anti-inflammatory effect [25,34,48]. Among the most critical structural elements that enhance the activity of AERs are chlorine or sulfate groups in the *N*-terminal residue [34,48], the presence of 6-OH-Choi in the third residue, D-allo-Ile in the second residue, or aminopiperidine/1-amidino-2-aminopyrrolidine (Amap/Aap) [33,49,50]. The presence of L-Argal moiety instead of D-Argal has also been mentioned [51]. Understanding the structure–activity relationship can help in the development of novel inhibitors with improved efficacy against specific molecular targets.

In addition to inhibitory activity against serine proteases and the potential application of AERs in the treatment of diseases induced by dysregulation of the enzymes (e.g., cancer or thrombosis), other activities of the compounds have also been explored [16,52,53]. AER828A has demonstrated anti-inflammatory activity in human hepatoma cell lines. They also induced the expression of cytochrome P450 enzymes, which are important in drug metabolism and detoxification [54].

## 4. Materials and Methods

### 4.1. Fractionation of Aphanizomenon Sp. KUCC C2 and Isolation of AER525

*Aphanizomenon* KUCC C2 (GenBank accession number OR987680), isolated from the Curonian Lagoon, was grown for biomass as previously described by Overlingė et al. [14]. The freeze-dried material (11 g) was extracted with 75% methanol (MeOH) in MilliQ water (280 mL × 2) by vortexing (15 min × 2), followed by centrifugation (4000 rpm, 20 min, 4 °C). The fractionation of the material was performed with the application of the Shimadzu HPLC system (Shimadzu Corporation, Kyoto, Japan). First, the sample was loaded onto a Biotage^®^SNAP KP-C18-HS flash chromatography column (120 g, 100 Å, 30 µm) (Biotage, Uppsala, Sweden) and a step gradient elution (15 mL × min^−1^) with a water–methanol mixture was applied. The collected fractions (45 mL) were evaporated in a centrifugal vacuum concentrator (MiVac, SP Scientific, Ipswich, UK) and analysed with the application of LC–MS/MS. Further separation of AER525 was performed in the Jupiter Proteo C12 preparative column (250 × 10 mm, 4 µm, 90 Å; flow rate 4 mL min^−1^) (Phenomenex, Aschaffenburg, Germany). The mobile phase was a mixture of MilliQ water (phase A) and 100% acetonitrile (phase B), both with the addition of 0.1% formic acid. To isolate pure AER525, the process was repeated several times using modified 30 min gradients starting from 0% phase B to 20% B. The fractions (1 mL) containing AER525 were combined and evaporated. The purity of the isolated AER525 (90–95%) was determined based on the LC–DAD chromatogram and the total ion chromatogram (LC–MS).

### 4.2. LC–MS/MS and HRMS Analysis

Fractions were analysed by LC–MS/MS system composed of Agilent 1200 HPLC (Agilent Technologies, Waldbronn, Germany) and a QTRAP5500 tandem mass spectrometer (Sciex, Toronto, ON, Canada). Compounds were separated in a Jupiter Proteo C12 column (4.6 × 150 mm; 4 μm; Phenomenex, Aschaffenburg, Germany) using a mobile phase composed of water with 0.1% formic acid (A) and acetonitrile with 0.1% formic acid (B). The gradient elution (0.4 mL min^−1^; 15 min) was from 0% B to 100% B. QTRAP5500 operated in the positive mode and the following conditions were used: ionisation voltage 5.5 kV, ion source temperature 550 °C, scan range 50–1000, declustering potential 80 eV, and collision energy 60 eV. The contents of the samples were determined in the IDA (information-dependent acquisition) mode; ions within the *m*/*z* range 300–1000 and intensity exceeding 5 × 10^5^ were detected and fragmented.

For the elucidation of the AER525 structure, high-resolution SYNAPT XS Q-TOF-MS (Waters, Milford, MA, USA) was used. The system was linked to Waters Acquity Premier LC equipped with a BEHC18 column (50 × 50 mm, 1.7 µm) (Waters, Milford, MA, USA). The mobile phase and gradient elution were the same as described above. SYNAPT XS Q-TOF-MS operated in positive ionisation mode under the following conditions: capillary voltage 3.0 kV, ion source temperature 150 °C, and trap collision energy range 10–80 V. For structural studies, MS^e^, MS/MS, and HDMSMS modes were used. In HD experiments, the transfer collision energy was 30–90 V. MasLynx 4.2 was used for data acquisition.

### 4.3. Enzymatic Assay

The enzyme inhibitory activity of fractions and AER525 was tested against trypsin [55], chymotrypsin [56], thrombin [56], elastase [57], and carboxypeptidase A [56]. For full details on trypsin, chymotrypsin, thrombin, and elastase inhibition assay refer to the work of Overlingė et al. [14,44]. In the carboxypeptidase A assay, the final concentration of the enzyme was 1.6 µM, *N*-(4-methoxy-phenyl-azoformyl)-Phe-OH (GLPBIO Technology LLC; Montclair, CA, USA) at a concentration of 0.2 mg mL^−1^ served as a substrate, and the carboxypeptidase inhibitor from potato tuber was used at a concentration range of 2.5–150 µg mL^−1^. Mixtures containing the sample (10 µL) or inhibitor (10 µL) were preincubated with the enzyme (10 µL) and buffer (160 µL; 50 mM Tris-HCl at pH 7.5) at 25 °C. After 10 min, the substrate (20 µL) was added, and the reaction mixture was incubated for another 10 min at 25 °C. The absorbance of the solutions was measured at 350 nm with the application of a microplate reader (Varioskan Flash, Thermo Fisher Scientific OY, Vantaa, Finland). All enzymes were from Sigma Aldrich (St. Louis, MO, USA); 1% *v*/*v* DMSO was used to dissolve fractions at final concentrations of 45 µg mL^−1^, while for pure AER525, it was additionally serially diluted to 45, 4.5, 0.45, and 0.045 µg mL^−1^. All tests were carried out in triplicates, and inhibition of enzyme activity by more than 50% was considered significant.

## 5. Conclusions

The study confirms that serine protease inhibition by *Aphanizomenon* sp. KUCC C2 extract is caused by a new AER variant AER525. Unfortunately, the tautomerisation of the *C*-terminal Argal moiety in the structure of AER525 significantly hindered the isolation of sufficient quantities of the peptide to perform the NMR analysis, reflecting similar difficulties faced by previous researchers. However, based on a rich fragmentation spectrum and measurements conducted with a high-resolution Time-of-Flight MS system, the structure of AER525 was suggested to be Ser* + Leu* + Choi + Argal. Here, Ser* and Leu* indicate the possible presence of structural isomers of the two amino acids which cannot be distinguished based on MS analyses. The results indicate that AER525 exhibits inhibitory activity against thrombin, suggesting its potential pharmaceutical applications. Further studies are needed to fully evaluate its potency and to investigate its effects on other proteases involved in the coagulation cascade. This report encourages further investigation of the mechanism of enzyme inhibition, alternative methods of AER525 production, and evaluation of the drug-like properties of the compound.

## Figures and Tables

**Table 1:** Fragments ions present in the AER525 spectrum collected with HRMS.

Fragment	Exact Mass	Calculated *m*/*z* [M+H]^+^	Observed *m*/*z*	Shift Mass
M	525.3275	526.3353	526.3359	Δ = 0.0006
M-H_2_O	507.3169	508.3247	508.3250	Δ = 0.0003
M+H-CH_3_N_2_-H_2_O	465.2951	466.3030	466.3006	Δ = 0.0024
M+H-Argal	368.21855	368.2185	368.2181	Δ = 0.0004
M+H-Argal-Choi	202.1317	203.1396	203.1550	Δ = 0.0154
(Argal-CH_3_N_2_)-H_2_O	96.0688	97.0766	97.0757	Δ = 0.0009
(Argal-CH_3_N_2_)+Choi	281.1740	282.1818	282.1824	Δ = 0.0006
(Argal-CN_2_H_5_)+Choi-H_2_O	265.1790	266.1869	266.1863	Δ = 0.0006
(Argal-CH_3_N_2_)+Choi+Leu	394.2580	395.2659	395.2732	Δ = 0.0073
M+H-Leu-Ser-H_2_O	307.2008	308.2087	308.2061	Δ = 0.0025
Leu+Ser-H_2_O	184.1212	185.1290	185.1311	Δ = 0.0021
C_3_H_8_N_2_O_2_	104.0586	105.0664	105.0798	Δ = 0.0134
Choi+Argal-NH_2_	308.1848	309.1927	309.1945	Δ = 0.0018
Choi+Argal-NH_2_-H_2_O	290.1743	291.1821	291.1848	Δ = 0.0027
Choi immonium		140.1075	140.1083	Δ = 0.0008

**Table 2:** The activity of AER525 against trypsin, thrombin, carboxypeptidase A, elastase, and chymotrypsin; “-” indicates not active (inhibition below 10%).

Compound	Enzyme Inhibition (IC_50_ [µM])				
	Trypsin	Thrombin	Carboxypeptidase A	Elastase	Chymotrypsin
AER525	71.7	0.59	89.7	-	-

## Data Availability

Data are contained within the article or Supplementary Material.

## Supplementary Materials

The following supporting information can be downloaded at https://www.mdpi.com/article/10.3390/md22110506/s1, Table S1: Ions detected in fractions 1–5. Fractions 2–4 showed activity against serine proteases. Bolded ions were present only in the active fractions. Table S2: Ions detected in fractions 23–26. Fractions 24 and 15 showed activity against serine proteases. The ion 526, corresponding to aeruginosine 525, has been bolded. Figure S1: Total Ion Chromatogram with peaks generated by peptides with identical fragmentation spectrum as AER525. Figure S2: Mass fragmentation spectrum of a compound with pseudomolecular ion at *m*/*z* 643. The spectrum contains a series of ions identical to AER525. Figure S3: Suggested structure of AER525 (A) and structure of dysinosin D (B).
