# Supplementary material for: Aeruginosin 525 (AER525) from Cyanobacterium Aphanizomenon Sp. (KUCC C2): A New Serine Proteases Inhibitor
Source: Mar Drugs. 2024 Nov 8;22(11):506. doi: 10.3390/md22110506 (PMC11595689; doi:10.3390/md22110506)
Supplement: Supplementary file 1 [file marinedrugs-22-00506-s001.zip › marinedrugs-3289377-supplementary.pdf]

Supplementary material

Aeruginosin 525 (AER525) from Cyanobacterium *Aphanizomenon* sp. (KUCC C2): A New Serine Proteases Inhibitor

Donata Overlingė [1,*], Marta Cegłowska [2], Robert Konkel [3], and Hanna Mazur-Marzec [3]

[1] Marine Research Institute, Klaipėda University, Universiteto av. 17, LT-92294 Klaipėda, Lithuania, donata.overlinge@ku.lt
[2] Institute of Oceanology, Polish Academy of Sciences, Powstańców Warszawy 55, PL-81712 Sopot, Poland; mceglowska@iopan.pl
[3] Department of Marine Biology and Biotechnology, University of Gdańsk, M. J. Piłsudskiego 46, PL-81378 Gdynia, Poland; hanna.mazur-marzec@ug.edu.pl; robert.konkel@ug.edu.pl
* Correspondence: donata.overlinge@ku.lt

Table S1. Ions detected in fractions 1-5. Fractions 2-4 showed activity against serine proteases. Bolded ions were present only in the active fractions.

Non active fraction F1			Active fraction F2			Active fraction F3			Active fraction F4			Non active fraction F5		
Mass	Time	Intensity	Mass	Time	Intensity	Mass	Time	Intensity	Mass	Time	Intensity	Mass	Time	Intensity
515	1.7	0.20	503	2.8	0.34	503	2.1	0.83	503	2.6	2.30	503	2.7	7.10
518	2.0	3.40	514	3.0	0.32	**508**	**1.8**	**0.24**	**508**	**2.2**	**0.28**	511	2.9	0.40
924	3.0	0.20	518	2.0	0.73	**529**	**2.6**	**0.30**	511	2.1	1.15	514	3.1	1.43
			524	2.2	0.28	**532**	**2.2**	**0.19**	**524**	**2.1**	**0.21**	530	2.0	1.06
			**542**	**3.0**	**2.50**	**537**	**1.9**	**0.25**	**529**	**3.1**	**2.40**	533	3.0	0.14
			**556**	**2.0**	**0.30**	542	3.0	10.8	542	2.8	1.46	542	2.4	0.94
			**562**	**2.7**	**0.50**	**553**	**1.6**	**0.26**	**562**	**3.1**	**0.42**	544	2.0	0.24
			**571**	**3,1**	**0.70**	**554**	**3.2**	**0.20**	564	2.5	0.35	564	3.0	0.90
			**579**	**2,3**	**0.91**	**562**	**2.9**	**1.68**	639	3.0	3.30	591	1.9	0.11
			**583**	**2.5**	**0.30**	**573**	**3.1**	**1.18**	**642**	**2.5**	**2.20**	593	2.5	0.33
			**623**	**2.2**	**1.34**	**654**	**2.3**	**0.40**	**680**	**3.0**	**1.60**	639	3.0	0.75

	**653**	**2.9**	**0.45**	**657**	**3.0**	**0.63**	**735**	**2.0**	**0.31**	1082	2.0	1.10
	**699**	**1.9**	**3.20**	**674**	**3.2**	**0.36**	**787**	**1.9**	**0.35**			
	**700**	**2.5**	**0.47**	**679**	**2.3**	**0.90**	**878**	**2.3**	**0.23**			
	**741**	**2.6**	**0.45**	**743**	**2.1**	**0.65**	**889**	**2.4**	**0.69**			
	**742**	**2.3**	**0.30**	**847**	**2.0**	**0.39**	**927**	**2.9**	**0.95**			
	**858**	**2.4**	**0.30**	**860**	**2.3**	**0.48**	1082	2.0	0.50			
	**872**	**2.4**	**0.38**	**889**	**2.3**	**1.73**						
	**875**	**2.4**	**0.42**	**911**	**2.8**	**0.25**						
	**889**	**2.7**	**1.08**	**943**	**2.9**	**0.25**						
	**900**	**1.9**	**0.90**	**1075**	**2.0**	**0.90**						
	**943**	**2.7**	**0.20**									
	**972**	**2.9**	**0.30**									
	**1075**	**2**	**0.59**									

Table S2. Ions detected in fractions 23-26. Fractions 24 and 15 showed activity against serine proteases. The ion 526, corresponding to aeruginosine 525, has been bolded.

Non active fraction F23			Active fraction F24			Active fraction F25			Non active fraction F26		
Mass	Time	Intensity	Mass	Time	Intensity	Mass	Time	Intensity	Mass	Time	Intensity
**526**	**2.0**	**0.35**	520	3.2	1.90	**526**	**2.3**	**45.80**	**526**	**2.3**	**12.1**
558	2.0	0.50	**526**	**2.3**	**38.70**	586	3.1	1.0			
616	2.5	0.55	564	2.0	8.2	616	2.8	2.2			
654	3.1	1.10	572	2.7	16.4						
			586	2.3	0.3						
			654	2.3	3.3						
			658	3.0	5.6						
			668	9.7	4.6						
			1029	9.6	1.4						

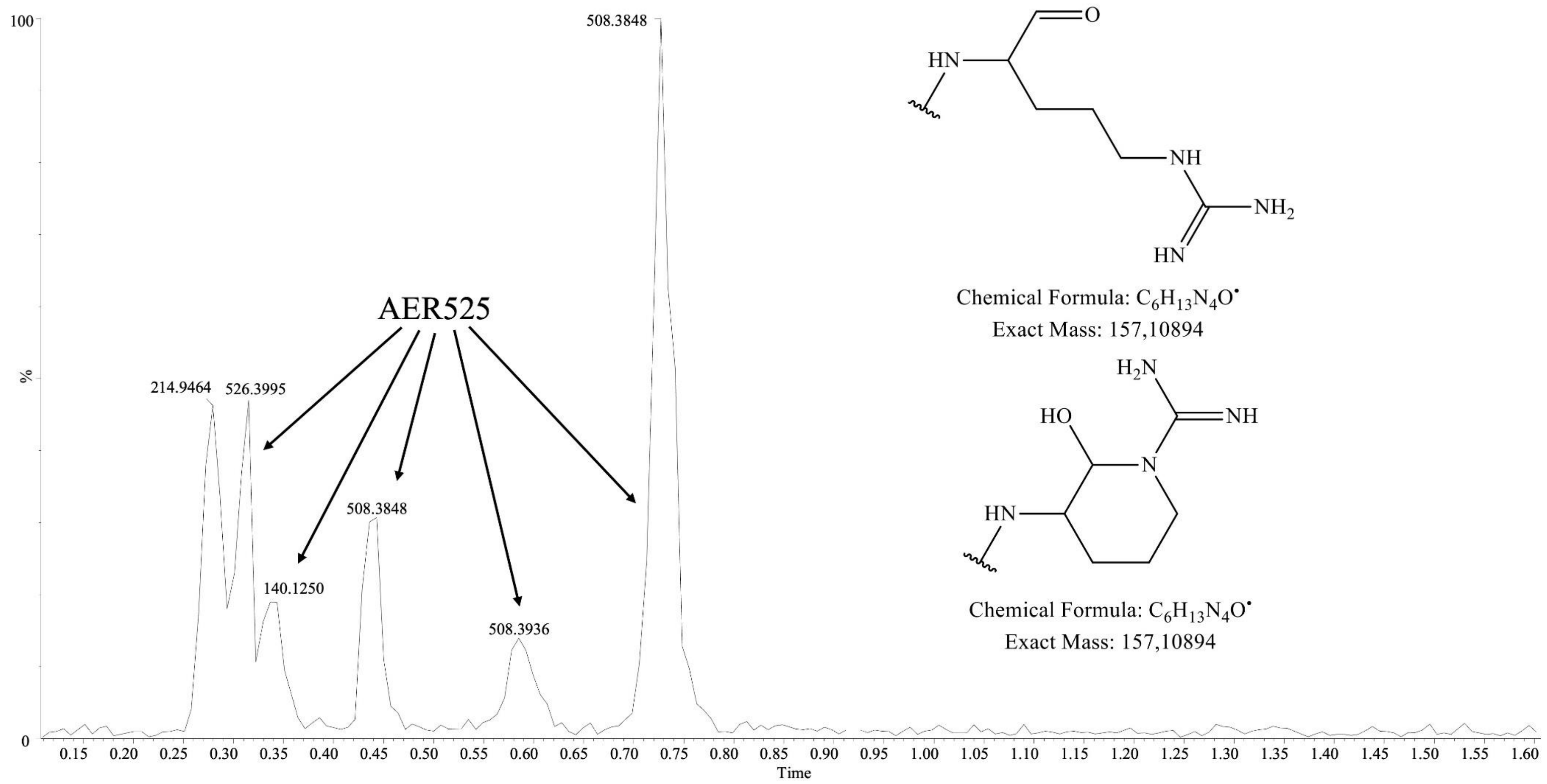

Figure S1. Total Ion Chromatogram with peaks generated by peptides with identical fragmentation spectrum as AER525.

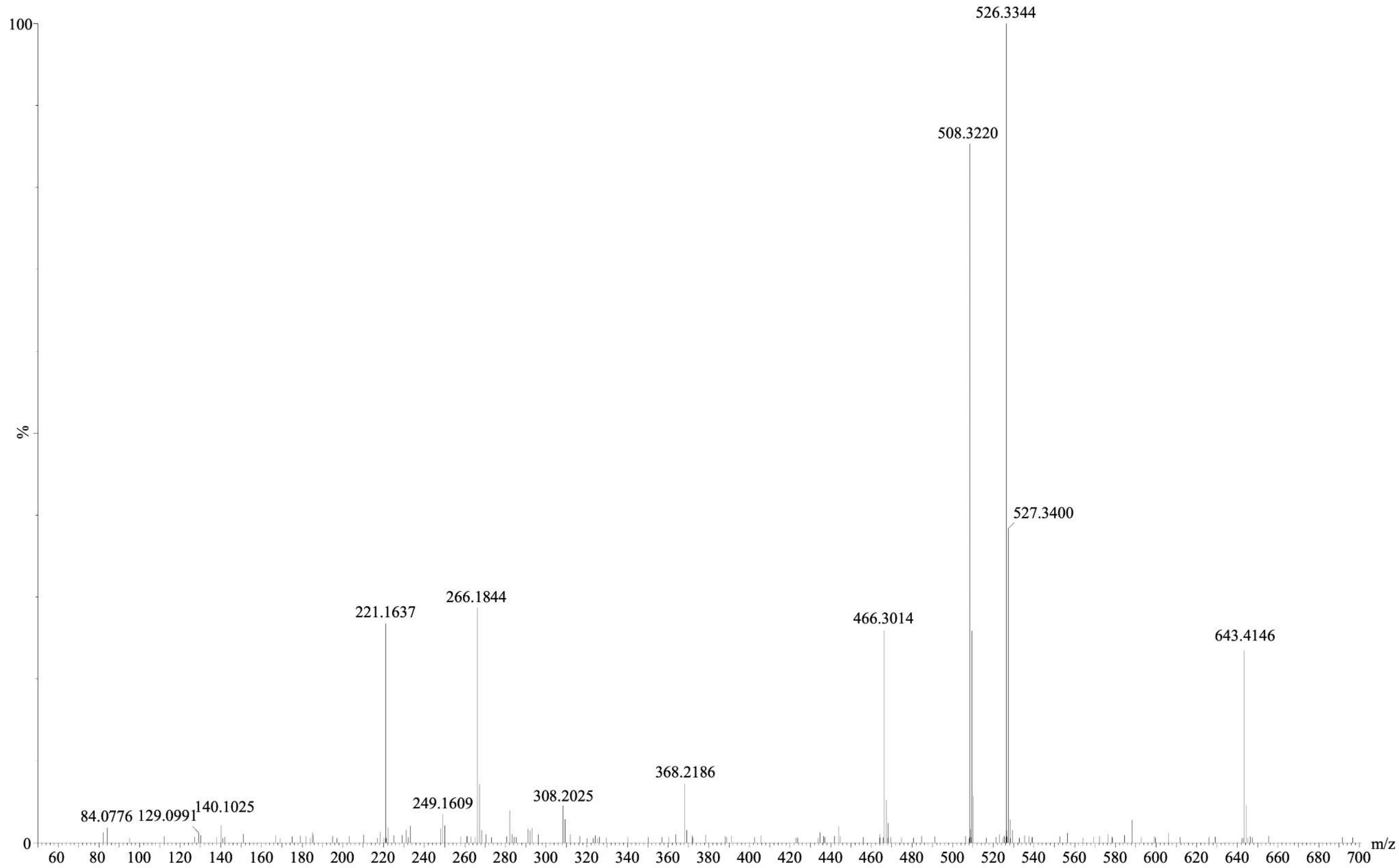

Figure S2. Mass fragmentation spectrum of compound with pseudomolecular ion at *m*/*z* 643. The spectrum contains a series of ions identical to AER525.

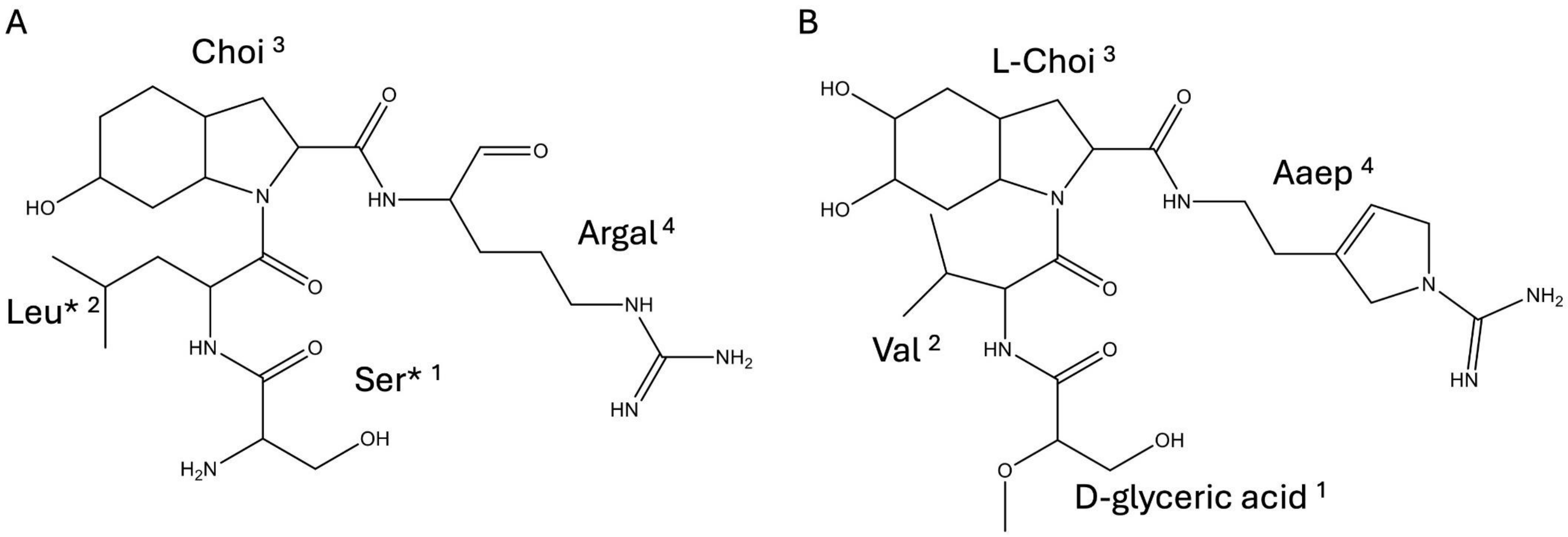

Figure S3. Suggested structure of AER525 (A) and structure of dysinosin D (B) (according to Carroll et al. [23]).
